# Cognitive and Neuropathophysiological Outcomes of Gamma-tACS in Dementia: A Systematic Review

**DOI:** 10.1007/s11065-023-09589-0

**Published:** 2023-03-06

**Authors:** Valerio Manippa, Annalisa Palmisano, Michael A. Nitsche, Marco Filardi, Davide Vilella, Giancarlo Logroscino, Davide Rivolta

**Affiliations:** 1 Department of Education, Psychology and Communication, University of Bari “Aldo Moro”, Bari, Italy; 2 Department of Psychology and Neurosciences, Leibniz Research Centre for Working Environment and Human Factors, Dortmund, Germany; 3 Department of Neurology, University Medical Hospital Bergmannsheil, Bochum, Germany; 4 Center for Neurodegenerative Diseases and the Aging Brain, University of Bari “Aldo Moro” at Pia Fondazione “Cardinale G. Panico”, Tricase, Lecce, Italy; 5 Department of Basic Medicine, Neuroscience and Sense Organs, University of Bari “Aldo Moro”, Bari, Italy

**Keywords:** Transcranial Alternating Current Stimulation, Non-invasive brain stimulation, Alzheimer’s Disease, Mild Cognitive Impairment, Neuromodulation, Neurodegenerative Diseases

## Abstract

Despite the numerous pharmacological interventions targeting dementia, no disease-modifying therapy is available, and the prognosis remains unfavorable. A promising perspective involves tackling high-frequency gamma-band (> 30 Hz) oscillations involved in hippocampal-mediated memory processes, which are impaired from the early stages of typical Alzheimer’s Disease (AD). Particularly, the positive effects of gamma-band entrainment on mouse models of AD have prompted researchers to translate such findings into humans using transcranial alternating current stimulation (tACS), a methodology that allows the entrainment of endogenous cortical oscillations in a frequency-specific manner. This systematic review examines the state-of-the-art on the use of gamma-tACS in Mild Cognitive Impairment (MCI) and dementia patients to shed light on its feasibility, therapeutic impact, and clinical effectiveness. A systematic search from two databases yielded 499 records resulting in 10 included studies and a total of 273 patients. The results were arranged in single-session and multi-session protocols. Most of the studies demonstrated cognitive improvement following gamma-tACS, and some studies showed promising effects of gamma-tACS on neuropathological markers, suggesting the feasibility of gamma-tACS in these patients anyhow far from the strong evidence available for mouse models. Nonetheless, the small number of studies and their wide variability in terms of aims, parameters, and measures, make it difficult to draw firm conclusions. We discuss results and methodological limitations of the studies, proposing possible solutions and future avenues to improve research on the effects of gamma-tACS on dementia.

## Introduction

Dementia refers to a spectrum of disorders characterized by neurodegenerative processes associated with progressive impairment of cognition and changes in mood and behavior that interfere with daily life. Approximately 40 million people worldwide suffer from Alzheimer’s disease (AD), the most common form of dementia. However, taking into account the rapidly increasing life expectancy, it is likely that the prevalence of AD will duplicate within the next 20 years (World Alzheimer Report, 2015). Despite the numerous pharmacological agents targeting dementia-related cognitive and behavioral symptoms, no disease-modifying therapy is currently available, and the prognosis remains unfavorable. Indeed, the available treatments are not effective in mitigating the neurocognitive disruption caused by the complex neuropathological cascade of AD involving microglia dysfunctions, formation of extracellular plaques of amyloid-beta peptides (Aβ), and intracellular tangles of hyperphosphorylated tau protein (p-Tau) (Affoo et al., 2013).

Over the last decade, the development of reliable disease biomarkers (Jack et al., 2016) has prompted interest in the preclinical stages of dementia (Dubois et al., 2016). AD is now conceptualized as a multidimensional process that progresses from *preclinical* (cognitively unimpaired people with biochemical markers of AD) to *clinical* phases (Jack et al., 2018). Particularly, a transitional phase separating normal aging from dementia known as Mild Cognitive Impairment (MCI) offers clinicians and researchers a window of opportunity for novel treatments. MCI is an umbrella term used to characterize the prodromal phases of dementia in which cognitive decline is faster and more pronounced than expected for age and educational level, albeit without significant impairment of everyday life and autonomy (Winblad et al., 2004). MCI can be categorized according to the specific features of cognitive impairment (amnestic MCI versus non-amnestic MCI), the number of impaired cognitive domains (single-domain MCI versus multiple-domain MCI) (Petersen, 2004), as well as the underlying neuropathological mechanism, such as MCI due to AD (MCI-AD; Albert et al., 2011). Overall, 10 to 15% of individuals with MCI are diagnosed with dementia within a year (Cooper et al., 2015), and 60% within five years (Gauthier et al., 2006). Since MCI is not inevitably prodromal of manifest neurocognitive disorders, the search for potential treatments to improve the prognosis of MCI is of utmost importance. To date, there are no effective treatments to prevent conversion from MCI to dementia (Cooper et al., 2013), likely due to the complex and multifactorial neuropathological process involved.

A promising perspective in this field involves the study of *brain oscillations* or brain waves, the coordinated activity of neural ensembles ranging from ⁓1 to ⁓120 Hz (Fries, 2005; Uhlhaas et al., 2010). Electroencephalographic (EEG) recordings during the resting state (rs-EEG) in AD patients indicate a global increase in the power spectral density of low-frequency oscillations (i.e., delta and theta-band activity ranging between 0.5⁓3.5 and 4⁓7.5 Hz, respectively) and a reduction and slowing of posterior alpha and beta activity (ranging between 8⁓13 Hz and 13⁓30 Hz, respectively), compared to healthy age-matched controls. Generally, the earliest detectable changes involve an increase in theta activity and a decrease in beta activity, followed by a decrease in alpha activity and an increase in delta activity, which are associated with both neuropathological markers and cognitive decline (Jafari et al., 2020; Jeong, 2004; Ranasinghe et al., 2022). Together with the disruption of functional brain connectivity, the enhancement of slow oscillations and the reduction of fast oscillations represent a possible marker of AD-related decline. Particularly, there is much evidence of gamma-band (> 30 Hz) disruption in AD patients (Jafari et al., 2020). Reduced gamma-band power accompanied by spontaneous gamma synchronization has been observed in both patients with AD and rodent models (Verret et al., 2012; Gillespie et al., 2016). Such high-frequency oscillations are of great interest due to their key role in information processing, higher cognitive functions, and cross-frequency synchronization underlying these processes (Başar et al., 2013; Sun et al., 2013). In particular, theta-gamma coupling is associated with memory encoding and retrieval processes mediated by the entorhinal and hippocampal structures (Mormann et al., 2005; Schack et al., 2002; Vivekananda et al., 2021). Moreover, a progressive increase of theta over gamma oscillations has been reported in MCI and dementia (Moretti et al., 2011; Musaeus et al., 2020).

Gamma-band activity refers to cortical oscillations primarily generated from the interaction between fast-spiking gamma-aminobutyric acid (GABA)-ergic inhibitory interneurons (i.e., parvalbumin-positive (PV^+^) interneurons), and excitatory pyramidal cells (Grent-‘t-Jong et al., 2018; Rivolta et al., 2015; Uhlhaas & Singer, 2012). The gamma-band range is conventionally divided into slow (30⁓50 Hz) and fast (55⁓120 Hz) oscillations. Narrow slow gamma frequencies ranging about 38⁓42 Hz are of particular interest for dementia because of their prominent involvement in hippocampal-mediated memory processes (Kirwan & Stark, 2004), usually impaired since early AD stages (Hampel et al., 2008). Moreover, abnormal narrow gamma-band activity is proportional to cognitive impairment (Babiloni et al., 2016; Koenig et al., 2005). This rhythmic aberration likely originates from the loss of GABAergic inhibitory interneurons and cholinergic innervation, caused by the accumulation of extracellular Aβ and tau-associated pathology (Mably & Colgin, 2018; Palop et al., 2007; Stam et al., 2002).

Starting from these observations, a seminal work in mice models of AD demonstrated that optogenetic and visual flicker stimulations at 40 Hz result in a marked reduction of extracellular Aβ aggregates and p-Tau tangles, potentially due to improved uptake of these proteins by microglia (Iaccarino et al., 2016). Similar results were obtained via electrical stimulation in a mouse model of AD (Zarifkar et al., 2019). Furthermore, recent evidence in mice showed that optogenetic enhancement of gamma-band activity leads to cortical vasodilatation and increases blood oxygenation (Mateo et al., 2017). These findings have led to preclinical human studies using non-invasive brain stimulation (NIBS), and particularly transcranial Alternating Current Stimulation (tACS), a neuromodulation technique that applies a low-intensity sinusoidal electrical current to the brain through surface electrodes placed over the scalp (Wischnewsk et al., 2022; Gonzalez-Perez et al., 2019). This approach allows the entrainment of endogenous cortical oscillations in a frequency- and phase-specific manner by modulating the membrane potentials (Elyamany et al., 2021; Jeong et al., 2021). Gamma-tACS (γ-tACS) is a suitable approach to replicate, in humans, the promising effects seen in murine models of AD (McDermott et al., 2018; Menardi et al., 2021). However, differences in stimulation parameters (i.e., duration, number of sessions, electrodes placement, current density), as well as different outcomes, render the interpretation of the available findings a challenging task. As such, this systematic review addresses the use of gamma-tACS in both MCI and dementia (mainly AD) patients to shed light on its feasibility, therapeutic impact, clinical effectiveness, and to suggest adjustments and future perspectives.

## Methods

This review was conducted according to the Preferred Reporting Items for a Systematic Review and Meta-analysis of Diagnostic Test Accuracy Studies (PRISMA-DTA) statement (McInnes et al., 2018). Following the PRISMA guidelines, two authors (VM and AP) conducted all methodological steps in the review process independently.

### Search Resources

The literature search was conducted using Pubmed (https://pubmed.ncbi.nlm.nih.gov/) and Web of Science (https://www.webofscience.com/) on reports published only in English language without limits of timeframe (last updated 15th August 2022). Search terms (keywords) included (a) *transcranial alternating current stimulation* or *tACS*, AND (b) *dementia*, *mild cognitive impairment* or *Alzheimer’s disease*. Following the identification of respective publications, we additionally screened the reference lists of the included studies to identify additional studies.

### Eligibility Criteria and Study Selection

**Fig. 1:**
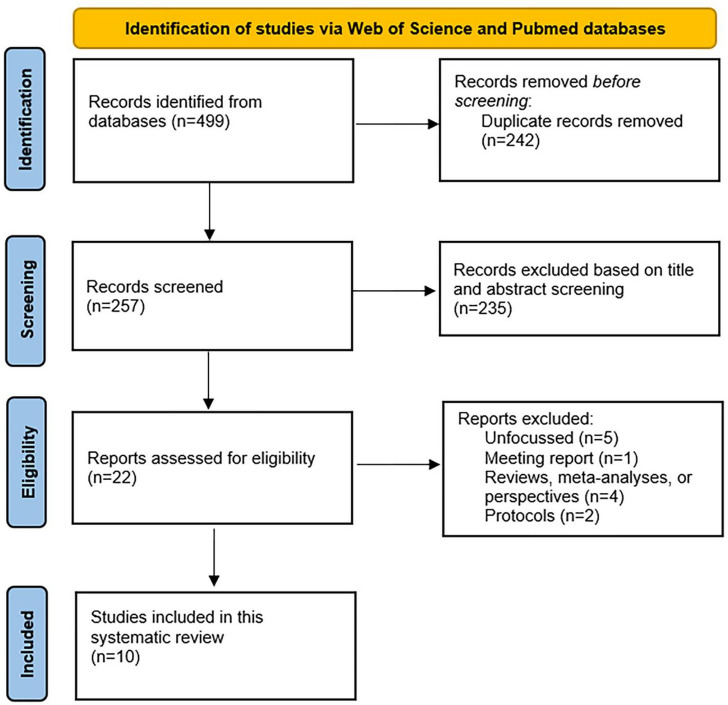
PRISMA flowchart illustrating the search and selection process of the papers included in this systematic review. In the eligibility stage, “Unfocussed” refers to reports using animal models of AD (n = 1), non-dementia samples (n = 1), tACS with non-gamma frequencies (n = 2), or sensory stimulation (n = 1)

The literature search yielded 499 results. After duplicate removal the remaining reports were screened for eligibility. Only original research (e.g., sham-controlled studies, open-label studies, pilot studies, clinical trials) that used gamma-tACS in MCI, AD, or other dementia patients were included in this systematic review (see the complete flowchart in Fig. 1). The high heterogeneity in tACS parameters, outcomes, study designs, and patients’ characteristics favored the choice of a narrative systematic framework for this review according to the guidelines by Popay et al. (2006). To organize the main results, the selected studies were arranged into single- and multi-session studies, and within this framework, detailed study characteristics were discussed.

### Data Extraction and Risk of Bias

The extracted data included clinical, methodological, and technical aspects. Since no standardized criteria or tools for the quality assessment of neuromodulation studies are available, VM and AP evaluated the risk of bias independently to assess sample size, presence or absence of a control or placebo condition (e.g., sham stimulation), details in the description of the statistical analyses, blinding of participants or data collectors, and diagnostic criteria used for study inclusion (sample homogeneity). A study was rated as having a low risk of bias when the sample size was sufficiently high, a control condition was defined, a detailed description of the statistical analysis was given, the experiment was conducted in a double-blind mode, and the sample was homogeneous. The risk of bias was rated as unclear when at least one criterion was not met, and as high when at least three criteria were not met.

## Results

Ten studies (273 patients analyzed) published between 2016 and 2022 were included in this review (Table 1). Four studies used single-session gamma-tACS, and six used multi-session gamma-tACS. The effects of gamma-tACS on cognitive functions or neuropathological markers were assessed for MCI (n = 2), mild-to-moderate AD (n = 5), unspecified MCI or dementia (n = 1), and mixed-diagnosis dementia patients (n = 1). One study comprised both MCI and AD patients. The outcomes were assessed offline (before and after tACS administration), except for two studies (Benussi et al., 2021, 2022), in which a cognitive task was administered during tACS and another immediately after. Five studies also assessed the feasibility of gamma-tACS treatment in terms of compliance (i.e., the number of participants that completed the study, or the dropout rate of the session), safety, and tolerability (self-reported serious and minor side effects, respectively). Study protocols and results are summarized in Tables 1 and 2.

**Table 1:** Designs and stimulation parameters of studies investigating the effects of gamma-tACS in mild cognitive impairment or dementia. N/A = not available

Study (year)	Sample	Experimental design	Research aim		Stimulation parameters					Risk of Bias
				Type of stimulation	Number of sessions	Duration (per session)	Electrode or coil location(s)	Stimulation intensity (peak-to-peak)	Electrode size	
Naro et al. (2016)	25 MCI 35 AD 27 HC	Crossover sham-controlled double-blind study (each participant was administered with different protocols	Testing the effect of gamma-tACS on cognitive functioning and the possibility to predict the MCI-to-AD conversion by exploring the gamma-tACS effects on brain oscillation	Gamma-tACS (continuously and randomly ranging from 40 to 120 Hz) in different left areas and sham tACS	Single	10 min	Active: M1 (C3), dlPFC (AF3-AF7), dmPFC (AF3-F1), PMA (FC3) or SMA (FCz) of the left hemisphere + sham stimulation C3-C4; Reference: right mastoid	1 mA	Active: 25 cm2; Reference: 35 cm2	Unclear
Kim et al. (2021)	20 MCI	Crossover sham-controlled double-blind study	Identifying the most promising and efficient therapeutic option between tACS and tDCS for cognitive enhancement in MCI patients	40 Hz-tACS, tDCS, and sham	Single	30 min	Bilateral dlPFC (F3-F4)	2 mA	28.26 cm2	Low
Benussi et al. (2021)	20 MCI-AD	Crossover sham-controlled double-blind study	Testing the safety/feasibility of 40 Hz-tACS applied over precuneus and its effect on memory performance and cholinergic transmission in MCI due to AD	40 Hz-tACS and sham tACS	Single	60 min	Active: Precuneus (Pz); Reference: right deltoid muscle	3 mA	5.5 × 6 cm	Low
Benussi et al. (2022)	60 AD (main study) 24 AD (control studies)	Crossover sham-controlled double-blind study	Testing the safety and predictors of 40 Hz-tACS effects on episodic memory, cholinergic transmission, and brain rhythms in early AD	40 Hz-tACS and sham tACS	Single	60 min	Active: Precuneus (Pz); Reference: right deltoid muscle	3 mA	5.5 × 6 cm	Low
Kehler et al. (2020)	17 MCI and dementia	Between-groups sham-controlled (parallel) study	Testing the effect of 4-week of brain exercises combined or not with 40 Hz-tACS on cognitive performance of MCI and dementia patients	Brain exercises + 40 Hz-tACS (n = 11); Brain exercises + sham tACS (n = 6).	20 sessions: 2 daily sessions (30 min each separated by 30 min break) for 4 weeks (5 days per week)	30 + 30 min	Active: left dlPFC; Reference: contralateral supraorbital area	1.5 mA	N/A	High
Zhou et al. (2021)	50 AD	Between-groups sham-controlled (parallel) double-blind study	Testing the safety/feasibility and the clinical effects (in terms of cognitive function and Aβ levels) of 40-Hz tACS in AD patients	40 Hz-tACS or sham tACS	30 sessions: 1 daily session for 6 weeks (5 days per week)	20 min	Bilateral temporal lobes	2 mA	4 × 4 cm	Low
Sprugnoli et al. (2021)	15 AD	Open-label studies	Testing the safety/feasibility and the effects of multi-session 40 Hz-tACS on cerebral perfusion, cognitive performance and rsEEG activity in mild to moderate AD patients	40 Hz-tACS	Group 1 and 2 = 10 sessions; Group 3 = 20 sessions (1 daily session 5 days per week for respectively 2 or 4 weeks)	60 min	Group 1: 8 circular electrodes on right temporal-frontal lobes (T8); Group 2 and 3: 4 circular electrodes on bilateral temporal lobes (P8, T8, P7, T7)	4 mA	3.14 (X 8 or X 4) cm2	High
Bréchet et al. (2021)	2 AD	Pilot study	Testing the safety /feasibility and effects of home-based 40 Hz-tACS system that could be used as an intensive neurorehabilitative treatment for dementia	40 Hz-tACS	70 sessions: 1 daily session for 14 weeks (5 days per week)	20 min	6 circular electrodes targeting the left angular gyrus (BA39/40)	4 mA	3.14 (X 6) cm2	High
Moussavi et al. (2021)	28 various dementia	Between-groups (parallel) study	Testing the effects of cognitive training program associated or not with a simultaneous 40 Hz-tACS on cognitive symptoms of dementia	Cognitive training + 40 Hz-tACS (n = 9); Cognitive training (n = 19)	20 sessions: 2 daily sessions (30 min each) for 4 weeks (5 days per week)	30 + 30 min	Active: left dlPFC; Reference: contralateral supraorbital area	1.5 mA	N/A	High
Dhaynaut et al. (2022)	4 AD	Pilot study	Testing the safety or feasibility and effects of 20 sessions of 40 Hz-tACS on Aβ deposition, p-Tau level, microglia activity, and gamma band activity in patients with mild to moderate AD	40 Hz-tACS	20 sessions: 1 daily session for 4 weeks (5 days per week)	60 min	Bilateral temporal lobes	4 mA	N/A	High

**Table 2:** Outcomes and main results of studies investigating the effects of gamma-tACS in mild cognitive impairment or dementia. N/A = not applicable

Authors (year)		Study outcomes			Main findings
	Neuropsychological	Electrophysiological	Neuropathological biomarkers	Assessment timing	
Naro et al. (2016)	Mini-Mental State Examination (MMSE), Reversal Motor Learning, Digit Span Forward, Clock Drawing Test, Attentive Matrices, Phonemic Letter Fluency, Verbal Fluency, Activities of Daily Living (ADL), Instrumental-ADL	rs-EEG	N/A	T0: Baseline; T1: Post-treatment; T1 + 60: 60 min after the treatment; T2: 2-years follow-up	The majority of MCI responded to dmPFC and dlPFC gamma-tACS with cognitive and electrophysiological improvements at T1 and T1 + 60. Non-responders’ MCI converted into AD at the T2 follow-up. AD outcomes were not influenced by any tACS protocol at any times
Kim et al. (2021)	Stroop test, Trial Making Test	rs-EEG	N/A	Baseline; Post-treatment	40 Hz-tACS improved the Stroop test in comparison with tDCS and sham and enhanced the TMT-B in comparison with sham. From EEG analysis, 40 Hz-tACS increased beta activity in comparison with sham and tDCS. 40 Hz-tACS also increases beta activity in the anterior cingulate cortex compared with sham. Minor effects were observed also after tDCS
Benussi et al. (2021)	Ray Auditory Verbal Learning Test (RAVLT), Face-Name Association Task (FNAT)	Cholinergic transmission (SAI assessed through TMS)	N/A	Baseline; Post-treatment	No tACS-related side effects were observed, and all participants well tolerated the intervention. A significant improvement in the RAVLT total recall, long-delayed recall scores, and FNAT scores was found following 40 Hz-tACS but not sham. Further SAI significantly increased only after 40 Hz-tACS
Benussi et al. (2022)	RAVLT, FNAT	Cholinergic transmission (SAI assessed through TMS), rs-EEG	Apolipoprotein E (ApoE) genotype	Baseline; Post-treatment	Results from Benussi et al. (2021) were replicated. In addition, a significant increase in gamma and beta power, a decrease in theta power, and a correlation between cognitive improvement and gamma power increase were found. ApoE genotype and cognitive impairment were the best predictors of 40 Hz-tACS response. Finally, two supporting studies demonstrated that the effects of 40 Hz-tACS were site- and cognitive function-specific
Kehler et al. (2020)	Wechsler Memory Scale (WMS), Montgomery Asberg Depression Rating Scale (MADSR)	N/A	N/A	T0: Baseline; T1: Post-treatment; T2: 1-month follow-up	Both groups significantly improved at the WMS from T0 to T1. At T2 this improvement was maintained better in the 40 Hz-tACS group compared to sham. No significant change in depressive symptoms was observed
Zhou et al. (2021)	MMSE, Alzheimer’s Disease Assessment Scale-Cognitive Subscale (ADAS-Cog)	N/A	Aβ (ELISA)	T0: Baseline; T1: Post-treatment; T2: 12-weeks follow-up	40 Hz-tACS improved MMSE from T0 to T1 and T2 and ADAS-Cog from T0 to T1. The ratio of Aβ 40:42 significantly decreased from T0 to T1 in the 40 Hz-tACS group. A significant correlation was found between the change in Aβ ratios and the total ADAS-Cog scores from T0 to T1. No safety issues were reported by the sample
Sprugnoli et al. (2021)	ADAS-Cog, MMSE, Montreal Cognitive Assessment (MoCA), Craft Story 21 Recall, Category Fluency task, ADL	rs-EEG	Perfusion-sensitive MRI	Baseline; Post-treatment	No adverse events were reported by participants. MRI revealed a significant increase in blood perfusion in the bilateral temporal lobes after the 40 Hz-tACS treatment. Moreover, perfusion changes displayed a positive correlation with changes in episodic memory and spectral power changes in the gamma-band
Bréchet et al. (2021)	MoCA, Memory Index Score	N/A	N/A	Baseline; Every 2 weeks; Post-intervention	The remotely monitored tACS intervention was found to be safe. Participants exhibited improvement in the cognitive tests completed every 2 weeks. Caregivers reported improvements in patients’ daily activities
Moussavi et al. (2021)	WMS, Egocentric Spatial Orientation, MADSR	N/A	N/A	T0: Baseline; T1: Post-treatment; T2: 1-month follow-up	Cognitive training significantly improved patients’ cognitive functions for one month after the intervention. At T2 40 Hz-tACS boosted the positive effect of cognitive training
Dhaynaut et al. (2022)	ADAS-Cog, MMSE, MoCA, ADL	rs-EEG	Aβ ([11 C]-PiB) p-Tau ([18 F]-FTP PET), and microglia ([11 C]-PBR28) through PET imaging	Baseline; Post-treatment	Participants tolerated the intervention without reporting any adverse events. An increase in gamma activity and a decrease of over 2% of p-Tau burden in 3 of 4 patients were observed after 40 Hz-tACS treatment. 1 of 4 patients reported a significant decrease in microglia activation whereas no change in the amount of intracerebral Aβ and cognitive performance were reported

### Single-Session Studies

The first study investigating tACS effects on MCI and AD was carried out by Naro et al. (2016). In this crossover sham-controlled repeated-measures study, 10 min of gamma-tACS randomly ranging from 40 to 120 Hz was administered in a sample of 25 MCI patients, 35 AD patients, and 27 healthy controls (HC). The tACS protocol was administered over the primary motor cortex (M1), premotor area, supplementary motor area, dorsolateral (dlPFC), and dorsomedial prefrontal cortex (dmPFC) within the left hemisphere. Sham-tACS was also administered over M1. The main aim of the study was to explore whether the effects of gamma-tACS on rs-EEG activity and cognitive functioning could identify MCI patients with a higher risk of AD conversion. MCI patients exhibited a significant cognitive improvement following dlPFC and dmPFC gamma-tACS, as compared to other protocols. These effects were identified in different cognitive tests including reversal motor learning (*p* = .03), digit span (*p* = .05), attentive matrices (*p* = .04), and letter verbal fluency (*p* = .03). Further, 21 MCI patients showed a general gamma-band oscillation increase immediately after stimulation and 60 min later (all *p*s < 0.004). On the other hand, four MCI patients did not show any significant tACS effect at baseline or 60 min later. At the two-year follow-up assessment, only the four non-responding MCI individuals were diagnosed with AD and showed a significant increase in gamma-band activity compared to baseline. No further cognitive and electrophysiological tACS effects were observed in the AD sample, while HC showed cognitive improvement and gamma-oscillation increase after gamma-tACS over prefrontal regions. The risk of bias was rated as unclear, mainly due to the non-exhaustive statistical reports.

Two further studies investigated the effects of a single-session gamma-tACS on MCI patients. In the sham-controlled, double-blinded, repeated-measures study of Kim et al. (2021), 20 MCI patients received both 40 Hz-tACS and tDCS administered over the dlPFC for 30 min. Cognitive tests, including the Stroop test and Trail-Making-Test (TMT) A and B, and EEG sessions were completed before and after single-session stimulation. Gamma-tACS improved performances on the Stroop test compared to both tDCS (*p =* .044, Cohen’s *d* = 0.81) and sham (*p =* .010, Cohen’s *d* = 1.13), as well as on the TMT-B compared to sham (*p =* .021, Cohen’s *d* = 0.33). Rs-EEG power analysis showed that gamma-tACS increased beta activity in right frontal regions compared to tDCS (*p <* .031, Cohen’s *d* = 1.00), and in right parietal regions compared to sham stimulation (*p <* .041, Cohen’s *d* = 0.54). Source analysis showed that 40 Hz-tACS also increased beta activity in the anterior cingulate cortex compared to sham (*p =* .005). Significant but minor effects on cognitive task performance and oscillatory activity were also observed after tDCS. The risk of bias was rated as low.

Benussi et al. (2021) assessed whether exposure to 40 Hz-tACS can improve memory and modulate cholinergic transmission in MCI due to AD. In this randomized, double-blind, sham-controlled, crossover pilot study, 20 participants were assigned to a single 60-minute session of 40 Hz or sham tACS targeting the precuneus. Memory performance was assessed offline using the Rey Auditory Verbal Learning Test (RAVLT), and online with the face-name association task (FNAT). Short-latency afferent inhibition (SAI), an indirect index of cholinergic transmission, was assessed offline using Transcranial Magnetic Stimulation (TMS). All participants completed the procedure without reporting tACS-related side effects or phosphenes. Participants were not able to distinguish between real and sham tACS, with cutaneous sensations equally perceived in both groups. Concerning cognitive outcomes, 40 Hz-tACS caused a significant improvement in RAVLT total recall performance compared to sham (*p <* .001, Cohen’s *d* = 0.73) and compared to baseline (*p <* .001, Cohen’s *d* = 0.73). Similarly, delayed recall performance was improved for 40 Hz-tACS compared to sham (*p =* .007, Cohen’s *d* = 0.70) and compared to baseline (*p <* .001, Cohen’s *d* = 0.66). 40 Hz-tACS also led to improved FNAT scores compared to sham (*p <* .001, Cohen’s *d* = 2.05). Congruently, SAI (i.e., cholinergic transmission) improved after gamma tACS compared to sham stimulation (*p <* .001, Cohen’s *d* = 2.39) and baseline (*p <* .001, Cohen’s *d* = 2.86). The risk of bias was rated as low.

Authors replicated these findings in a subsequent study with a sample of mild AD patients (Benussi et al., 2022). Sixty patients assigned to the main study received 60 min of 40 Hz tACS and sham tACS one week apart targeting the precuneus. In each session, RAVLT performance and SAI were assessed at baseline and after tACS session. Online FNAT was administered during stimulations. In addition, EEG analysis and individualized modeling of electric field distribution from MRI data were performed in a subset of patients. Finally, overall cognitive functioning assessed with the Mini-Mental State Examination (MMSE) and genetic predictors (Apolipoprotein E [ApoE] genotype) of gamma-tACS efficacy were evaluated. Authors confirmed previous results (Benussi et al., 2021): 40 Hz-tACS was well tolerated and improved RAVLT immediate recall performance compared to sham (*p <* .001, Cohen’s *d* = 0.93) and baseline (*p <* .001, Cohen’s *d* = 0.94), delayed recall performance compared to sham (*p <* .001, Cohen’s *d* = 0.88) and baseline (*p <* .001, Cohen’s *d* = 0.56), and FNAT performance compared to sham (*p <*. 001, Cohen’s *d* = 0.97). Cholinergic transmission also improved after 40 Hz-tACS compared to sham (*p <* .001, Cohen’s *d* = 2.44) and baseline (*p <* .001, Cohen’s *d* = 2.99). Two supporting control studies were conducted, each with 12 AD patients, and showed that the effect of 40 Hz-tACS was specific for memory performance compared to executive functions, verbal fluency, and visuospatial abilities, and occurred exclusively when the target of stimulation was the precuneus (versus the right dlPFC). Also, immediately after 40 Hz-tACS, both a significant gamma and beta power increase on posterior sites and a significant decrease in theta power on fronto-temporal sites were observed (all *p*s < 0.05). Particularly, memory improvement correlated with gamma frequency increase in parietal regions and with the amount of predicted electric field distribution in the precuneus. Finally, both baseline disease severity and ApoE genotype (i.e., the major recognized genetic risk factor for late-onset AD) were the best predictors of 40 Hz-tACS response. Risk of bias was rated as low.

**Fig. 2:**
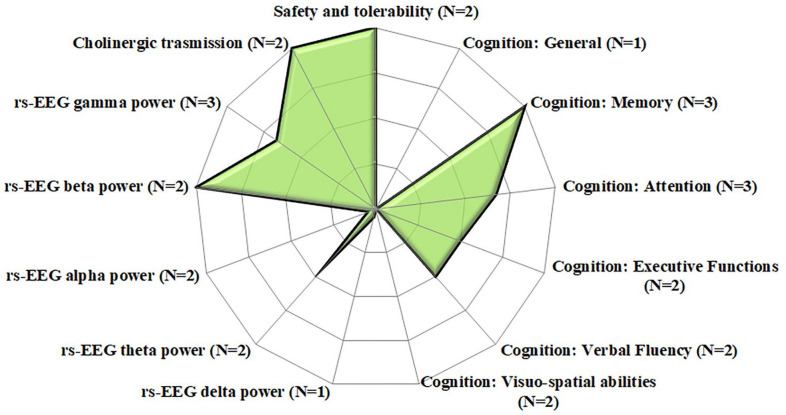
Profile plot on the effects of single-session gamma-tACS on different dementia outcomes. The plotted data represent the percentage of studies that found significant effects for each outcome ranging from 0 (i.e., the “blank” areas of the plot, which indicate that no studies found a significant effect on that outcome) to 100 (i.e., the green areas reaching the edge of the plot, which indicate that all studies found a significant effect on that outcome), N = total number of studies investigating each outcome

Overall, despite the variability in stimulation parameters, all studies found that gamma-tACS was well-tolerated and induced significant improvements in memory and attention performance. Two studies also reported tACS-induced changes in rs-EEG, with an increase in beta- and gamma-band power, and a decrease in theta-band power, as well as cholinergic transmission improvements (see Fig. 2). All single-session studies were sham-controlled gamma-tACS protocols.

### Multi-Session Studies

Moussavi et al. (2021) published a pilot study to explore the effects of cognitive training with and without 40 Hz-tACS in a heterogeneous sample of 28 dementia patients (Moussavi et al., 2021). The sample was divided into two non-randomized and non-matched groups depending on the participants’ preference to receive only cognitive training or cognitive training coupled with 40 Hz-tACS. The active electrode was placed over the left dlPFC, while the reference was positioned over the contralateral supraorbital area. The treatment was administered for 4 consecutive weeks, 5 days per week, and two 30 min-sessions per day. Patients’ cognitive functioning was assessed pre-intervention (T0), post-intervention (T1), and one month (T2) after the end of the intervention, using the Wechsler Memory Scale (WMS), Egocentric Spatial Orientation (ESO) test, and the Montgomery Asberg Depression Rating Scale (MADRS). Results showed significant improvements in WMS and ESO performance at T1 and T2, with a higher WMS improvement for those participants who received tACS combined with cognitive training, compared with those receiving cognitive training only (*p* = .043, Cohen’s *d* = 1.45). Finally, no effects emerged for the MADRS scores. The risk of bias was rated high because of the arbitrary sample selection and group arrangement, the lack of a control condition, and no statement on blinding.

Similarly, in a between-group study, Kehler et al. (2020) investigated the effectiveness of brain exercises combined or not with simultaneous tACS on cognitive functions in MCI and mild-to-moderate unspecified dementia patients. A total of 17 participants completed cognitive training during two 30-minute sessions per day, 5 days per week for 4 consecutive weeks. During cognitive training, 11 participants were stimulated with 40 Hz-tACS over the left dlPFC (with the reference over the contralateral supraorbital area), while the remaining participants received sham stimulation. WMS scores significantly improved in both groups between baseline and post-intervention (*p =* .04 for the sham plus brain exercises group, and *p <* .001 for the active tACS group). Further, at the one-month follow-up, the 40 Hz-tACS group maintained its improvement to a larger degree than the sham group (*p =* .049). The risk of bias was rated high because of the arbitrary sample selection and group arrangement, the lack of a control condition, and no statement on blinding.

Bréchet et al. (2021) developed a patient-tailored home-based tACS protocol, which was administered by trained caregivers and remotely monitored by the study team. The protocol consisted of 20 min of 40 Hz-tACS for 14 weeks, 5 days per week, resulting in 70 sessions per participant, via a wireless battery-driven current stimulator with six electrodes (two anodes and four cathodes) targeting the left angular gyrus (AG). This stimulation montage was defined using tACS-induced electric field modeling. The sample included two AD patients, while the non-visual version of the MoCA and Memory Index Score (Uniform Dataset of the National Alzheimer’s Coordinating Center) served as qualitative outcomes. This study demonstrated the feasibility and the safety of a remotely monitored, caregiver-administered, home-based tACS intervention. Both participant-administrator dyads showed 100% adherence to the protocol, missing none of the 70 scheduled sessions in the 14 study weeks. Only a few occurrences of headache and concentration difficulty were reported. No technical issue emerged and only two sessions were auto-aborted due to high impedance or internet connection cut-off. Concerning cognitive outcomes, participants exhibited improvement in the Memory Index Score and the non-visual version of the MoCA during the intervention and at the 3-month follow-up compared with baseline. In addition, their caregivers reported improvements in patients’ daily activities with fluctuations from day to day. Both caregivers reported improvements including a reduction in repetitive questions, better orientation, and superior recall of plans. The risk of bias was rated high due to the small sample size, the lack of a control condition and the predominant use of qualitative data.

Three studies considered neuropathological biomarkers as outcomes of a multi-session gamma-tACS treatment. Zhou et al. (2021) performed a between-group study involving 50 AD patients. Twenty-three participants underwent a 6-week 40 Hz-tACS intervention (20 min per day) over the bilateral temporal lobes, while the remaining participants received sham tACS. MMSE and Alzheimer’s Disease Cognitive Component Assessment (ADAS-Cog) were used for cognitive evaluation at baseline, at the end of the intervention (T1), and 12 weeks after treatment (T2). The Aβ 40:42 ratio was assessed from peripheral blood analysis. First, the safety of the protocol was confirmed. Moreover, in the tACS group, a significant improvement in MMSE scores was reported at T1 (*p* = .012) and T2 (*p =* .034) compared to baseline, whereas the ADAS-Cog scores improved only at T1 (*p =* .005). Regarding serum biomarkers, the ratio of Aβ 40:42 significantly decreased at T1 compared with baseline (*p =* .03) in the active gamma-tACS group only. Furthermore, significant correlations emerged between the change in Aβ 40:42 ratios and ADAS-Cog scores from baseline to T1 (r = .50, *p* = .015) in the active tACS group, particularly in verbal memory subtests. The risk of bias was rated as low.

One open-label study from Sprugnoli et al. (Sprugnoli et al., 2021) investigated tACS effects on cerebral blood perfusion (CBF) in 15 mild-to-moderate AD patients. One session of 40 Hz-tACS per day was administered for two or four weeks, primarily targeting the right frontotemporal or the bilateral temporal lobe. Perfusion-sensitive MRI scans were collected at baseline and right after the intervention, along with rs-EEG and cognitive assessments. All participants completed the study and tolerated the intervention although with minor side effects commonly associated with tACS stimulation (i.e., tingling, scalp irritation, visual changes, headache). Participants attended 95% of the study visits, showing excellent treatment compliance. Regarding the clinical outcomes, post-intervention CBF values in the right temporal lobe increased significantly (*p <* .05, Cohen’s *d* = 0.22). Further, 38–42 Hz narrow gamma (38–42 Hz) spectral power displayed a post-tACS increase higher than activity in the theta (*p <* .01, Cohen’s *d* = 0.31), beta (*p <* .05, Cohen’s *d* = 0.26), and high gamma (*p <* .05, Cohen’s *d* = 0.24) bands. Narrow gamma spectral power changes at the right temporal lobe correlated with the clusters of significant CBF increase (r = .57; *p =* .05). Finally, no significant cognitive improvements emerged after tACS. Despite that, significant CBF changes positively correlated with changes at Craft Story Recall - Delayed (r = .53, *p* = .04). The risk of bias was rated high due to the small sample size, and the lack of a control condition.

The most recent study from Dhaynaut et al. (2022) aimed to reduce Aβ and p-Tau deposition by modulating microglia activity in AD patients using tACS. Four participants with mild-to-moderate AD received daily 40 Hz-tACS for 1 h per day over 4 weeks (5 days per week) targeting the bitemporal lobes. Aβ, p-Tau, and microglia were assessed through PET imaging before and after the intervention along with electrophysiological assessments. Participants completed the study without reporting any adverse events and attended 95% of the study visits showing excellent treatment compliance. Qualitative analyses showed a post-treatment increase in EEG gamma spectral power, as well as a decrease of over 2% of p-Tau burden in three out of four patients, mainly involving the temporal-mesial regions. The amount of intracerebral Aβ was not significantly influenced by tACS, whereas one patient reported a significant decrease in microglia activation. The risk of bias was rated high due to the small sample size, the lack of a control condition, and the predominant use of qualitative data.

**Fig. 3:**
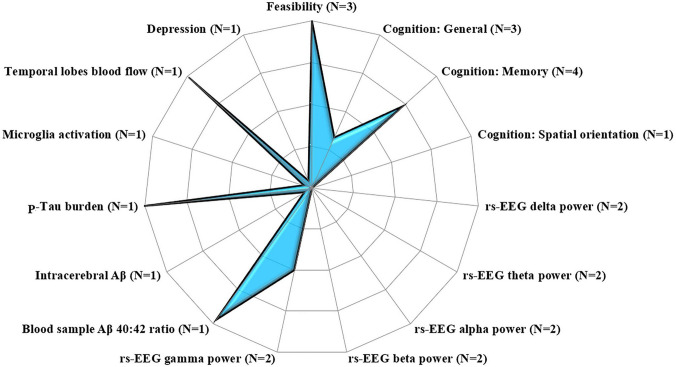
Profile plot on the effects of multi-session gamma-tACS on different dementia outcomes. The plotted data represent the percentage of studies that report significant effects on each outcome, ranging from 0 (i.e., the “blank” areas of the plots, which indicate that no studies found a significant effect on that outcome) to 100 (i.e., the blue areas reaching the edge of the plot, which indicates that all studies found significant effects on that outcome parameter). N = total number of studies investigating each outcome

Overall, three of the multi-session studies demonstrated the feasibility (in terms of patients’ compliance and lack of adverse effects) of multi-session 40 Hz-tACS interventions. The majority of the studies focused on mild-to-moderate AD, and some studies lacked a control condition (N = 3). Two studies were coupled with cognitive training programs. Despite the general high risk of bias and the variability of the intervention parameters, the majority of studies reported significant improvements in memory performances following interventions. Some of such improvements lasted for 1 to 2 months after the treatment. One study observed an increased rs-EEG gamma activity after treatment, while the other frequency bands were not affected by gamma-tACS. Findings about the effects on neuropathological biomarkers were more scattered and unequivocal (see Fig. 3).

## Discussion

This systematic review suggests the feasibility of gamma-tACS in MCI and dementia patients. All participants completed the respective protocols without showing serious adverse effects. Most of the reviewed studies showed cognitive improvement following gamma-tACS, while only trials with small samples reported non-significant effects on cognition. Although some studies showed promising effects on neurophysiological and neuropathological biomarkers, further research is needed to fill the gap with the strong evidence deriving from gamma optogenetic, sensory, and electrical stimulation in mouse models of AD. In addition, drawing firm conclusions is difficult due to the small number of studies and their wide variability in terms of aims, and stimulation parameters.

Concerning the single-session studies, evidence shows that 10 min of gamma-tACS, regardless of the site of stimulation (both frontal and parietal areas were tested), is sufficient to improve cognitive functioning and modulate brain oscillations acutely for at least 60 min after stimulation (Kim et al., 2021; Naro et al., 2016). Noteworthy, Naro et al. (2016) showed that MCI patients who did not show significant neuropsychological and electrophysiological effects after tACS were more likely to convert to AD within two years. Furthermore, two sham-controlled crossover protocols conducted by the same research group (Benussi et al., 2021, 2022) demonstrated that 60 min of 40 Hz-tACS over the precuneus can improve episodic memory, gamma and beta-band activities, and cholinergic transmission (assessed by SAI) in both MCI and AD patients. Particularly, ApoE genotype and disease stage were predictors of AD participants’ 40 Hz-tACS response, with greater cognitive improvement observed in ApoE ε4 noncarriers and earlier disease stage patients (compared to ApoE ε4 carriers and late disease stage patients). These observations may have important clinical implications considering that the disruption of gamma oscillations in AD patients is proportional to cognitive decline and loss of cholinergic innervation (Babiloni et al., 2016; Koenig et al., 2005). Furthermore, gamma abnormalities are mediated by the accumulation of Aβ and p-Tau tangles (Palop et al., 2007; Stam et al., 2002), in which the ApoE ε4 plays a key role (Mormino et al., 2014).

Concerning the multi-session tACS protocols, all the reviewed studies support the feasibility, safety, and tolerability of tACS treatments, with no adverse effects reported by any of the participants, despite the wide variability of stimulation parameters and protocols. Most studies showed cognitive improvements after tACS, especially in the domain of memory. However, the improvement was not always greater than that observed in the sham-tACS group (e.g., Zhou et al., 2021) and only a few studies assessed the long-term effects of the treatment. Increased hippocampal perfusion and gamma oscillation activity (Dhaynaut et al., 2022; Sprugnoli et al., 2021) have also been reported following gamma-tACS. Differential changes in Aβ and p-Tau deposition levels have been observed following tACS (Dhaynaut et al., 2022; Zhou et al., 2021). Particularly, Zhou et al. (2021) demonstrated that 30 sessions (20 min per day) of tACS bilaterally over the temporal lobes resulted in a significant decrease in the plasma Aβ 40:42 ratio. The protocol (20 sessions, one hour per day) from Dhaynaut et al. (2022) using PET imaging showed a reduction in p-Tau burden, but not in Aβ deposition.

The majority of the studies assessed tACS effectiveness on cognitive performance, and memory in particular, with positive results. The progressive decline in cognitive functioning is the hallmark of dementia, which in AD and MCI-AD usually starts in the domains of memory and particularly with the impairment in encoding and retention of new information (i.e., episodic memory). Congruently, MMSE, MoCA, or ADAS-Cog were usually used to measure global cognitive functioning outcomes. These tests have been specifically developed to detect AD-related cognitive impairment. Two studies (Kehler et al., 2020; Moussavi et al., 2021) adopted the WMS to evaluate memory impairment, while other studies adopted the RAVLT and the FNAT (Benussi et al., 2021, 2022), the Reversal Motor Learning (Naro et al., 2016), and the Craft Story 21 Recall Immediate and Delayed (Sprugnoli et al., 2021; Bréchet et al., 2021) tested the efficacy and feasibility of a patient-tailored home-based tACS protocol. A personalized montage was defined by using tACS-induced electric field modeling. AD patients reported a qualitative increase in the Memory Index Score (defined by the National Alzheimer’s Coordinating Center) assessed every 2 weeks during the treatment. The studies from Benussi et al. (2021, 2022) were the only that adopted two parallel versions of the RAVLT. To the best of our knowledge, no parallel versions are available for the ADAS-Cog, MMSE, and WMS.

Neurophysiopathological measures were also collected in various studies. A commonly used outcome is the power spectral analysis of rs-EEG, due to the available evidence for progressive reduction in beta, alpha, and gamma, in favor of higher theta and delta activity in dementia (Jafari et al., 2020; Jeong et al., 2021). The high-to-low-frequency ratio is significantly decreased in clinically diagnosed AD patients compared to healthy individuals, with MCI patients remaining in between (Moretti et al., 2011; Musaeus et al., 2020). The rationale underlying electrophysiological recordings in tACS studies is that 40 Hz stimulation in AD or MCI patients could entrain the reduced endogenous narrow gamma frequencies involved in memory processes, as well as modulate the activity in diverse cortical regions through brain networks resonance (Ali et al., 2013). However, this mechanism is still unclear, as a large proportion of neurons do not respond to tACS entrainment (Beliaeva & Polania, 2020; Johnson et al., 2020). Three single-session studies adopted rs-EEG spectral density power as an outcome. Naro et al. (2016) showed that both MCI and AD exhibit lower alpha and beta activity over the parieto-occipital lobes, enhanced parieto-occipital theta, delta, and gamma activities, and higher theta-gamma ratio (larger in AD than in MCI) compared with healthy controls. After 10 min of gamma-tACS over the dlPFC, a whole-brain increase of gamma-band activity emerged for up to 60 min after stimulation. Similar but more localized effects were found when tACS was applied over the dmPFC and M1. Also, a significant positive correlation emerged between gamma-band activity and performances at attentive matrices and clock drawing test, providing further support for the gamma-band implication in high-order processes. Finally, MCI patients showing no significant dmPFC-tACS after-effect (both neuropsychological and rs-EEG) converted to AD within two years. By contrast, patients who showed an increase in gamma oscillations did not convert to AD (Naro et al., 2016). This result might be relevant for future potential applications of combined tACS-EEG protocols for biomarker identification (e.g., perturbation-based markers), especially in terms of disease progression. Furthermore, Kim et al. (2021) and Benussi et al. (2022) reported gamma-tACS enhancement of beta and gamma activity, together with cognitive performances, as compared to both tDCS and sham conditions. Benussi et al. (2022) also found that the greater the increase in gamma power in posterior regions and in the electric field distribution in the precuneus, the greater the cognitive improvement was following 40 Hz-tACS.

The two multi-session studies that assessed rs-EEG features before and after gamma-tACS reported a specific increase in gamma spectral power after treatment. Particularly, while Sprugnoli and coworkers (2021) showed that narrow gamma spectral power changes over T8 (i.e., the right temporal lobe) correlated positively with hippocampal cerebral blood flow and with performances at the Craft Story Recall test (delayed), Dynahut et al. (2022) showed that each session of 40 Hz-tACS induced a gamma-band spectral power increase with a partial reset when stimulation was not administered. At the end of the intervention (1 h per day for 4 weeks), the increase of gamma spectral power was associated with a decrease in p-Tau burden over the stimulated bilateral temporal lobes. These promising results are consistent with those obtained via 40 Hz optogenetic and sensory entrainment in preclinical models of AD (Martorell et al., 2019).

Despite the outlined variability of intervention and outcome measures, the available data allow identifying converging evidence to guide future interventional avenues. First of all, multiple research groups agreed on the stimulation frequency. Despite gamma cortical oscillations ranging from ⁓30 to ⁓120 Hz, 40 Hz has been adopted in nine out of 10 studies. Only Naro et al. adopted continuous and random frequency stimulation ranging from 40 to 120 Hz (Naro et al., 2016). This study was published before the seminal work of Iaccarino et al. (2016), which demonstrated that 40 Hz light flicker reduces Aβ levels in a mouse model of AD, with no effects of other stimulation parameters or frequencies (i.e., constant light-on or dark, 20 Hz, 80 Hz, and random flicker). Since then, all studies investigating the effect of tACS on dementia-related cognitive impairment or biomarkers provided stimulation at a frequency of 40 Hz. The reviewed tACS studies also agree on parameters including current intensity, usually set at 2 mA, and the treatment duration for the multi-session tACS protocols (20 sessions, 1 h per day).

**Fig. 4:**
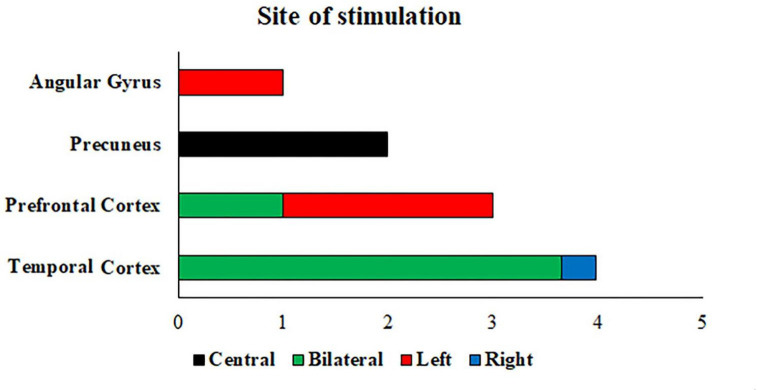
**The stimulation sites targeted by the reviewed studies, and the number of studies administering cognitive tasks or training during gamma-tACS.** Note: Naro et al., (2016) administered gamma-tACS in several left frontal and central regions, but the most effective targets for gamma entrainment and cognitive improvement were the prefrontal regions. Sprugnoli et al., (2021) administered gamma-tACS over the right (fronto-)temporal cortex in one group and over the bitemporal cortex in the other two groups

Regarding electrode placement, the most common and promising sites of stimulation are the temporal lobes, the dlPFC, and the precuneus (see Fig. 4). Indeed, the entorhinal-hippocampal circuit, situated in the temporal mesial lobe is the first region undergoing disruption during the early stage of AD (i.e., due to concomitant neuropathological processes involving hypoperfusion, Aβ, and p-Tau accumulation, and the consequent atrophy) (Hampel et al., 2008). These areas are strongly involved in memory encoding and retrieval (Kirwan & Stark, 2004) and exhibit prominent gamma oscillations that are usually dysregulated in both AD patients and murine diseased models (Babiloni et al., 2016; Verret et al., 2012). Hippocampal regions, dlPFC, and precuneus have extensive structural and functional mutual connections and play a pivotal role in brain functional integration (Elman et al., 2016; Grothe et al., 2016). Activity in these regions is typically associated with memory retrieval and formation, perceptual attention, decision-making, and executive functions (Sestieri et al., 2017; Wang et al., 2014). Interestingly, while three studies had personalized electrode placement using EEG current flow modeling (Benussi et al., 2021; 2022; Bréchet et al., 2021; Sprugnoli et al., 2021) investigated the effects of multi-session 40 Hz-tACS over the bitemporal lobes versus the right temporal-frontal lobes according to individual Aβ accumulation maps in individual patients. They found that improvements in memory performance, blood perfusion, and gamma activity were comparable regardless of the stimulation site. Given the interest tACS is gathering in the field of dementia, further methodological considerations are needed to develop effective approaches (for instance, the development of state-dependent stimulation protocols through concurrent tACS-EEG; Menardi et al., 2021; Guarnieri et al., 2020).

To date, the most frequently adopted gamma-tACS protocols consist of 20 sessions of 40 Hz-tACS (ranging 1.5–4 mA peak-to-peak) targeting the bilateral temporal lobes (1 h per day, distributed across 1 month). Accordingly, two ongoing protocols, registered on Clinicaltrials.gov (NCT04425148, NCT03920826), are currently investigating the effect of 30 one-hour sessions of 40 Hz-tACS on Fronto-Temporal Dementia (GIFTeD trial) and AD patients (TRANSFORM-AD trial), respectively (Assogna et al., 2021; Xing et al., 2020). Another ongoing protocol on dementia patients (Jacobson et al., 2022; NCT05203523) is evaluating the short- and long- term benefits of 40 Hz-tACS treatment coupled with cognitive training compared to sham tACS (4 weeks, 5 days per week, in two 30 min daily sessions).

### Proposed Mechanisms of Action of Gamma-tACS

Gamma brain oscillations result from resonant interaction between inhibitory GABAergic interneurons and excitatory (glutamatergic) neurons (Cardin et al., 2009; Sohal et al., 2009). The current models suggest that rhythmic synaptic inhibition regulates the spiking activities of neurons in local neurocircuits during gamma oscillations (Buzsáki & Wang, 2012; Sohal, 2016). If rhythmic interactions between inhibition and excitation generate gamma oscillations, then it would be possible to induce or modulate gamma by changing inhibition or excitation at specific temporal patterns (Adaikkan & Tsai, 2020). Optogenetic studies showed that gamma stimulation can increase large-scale PV + interneuron activity (i.e., from cortical areas to deeper brain regions such as the hippocampus) (Catani et al., 2003; Kanwisher & Dilks, 2013), improve the excitatory-inhibitory (E-I) imbalance observed in AD patients (Chan et al., 2021; Hijazi et al., 2020), and enhance communication among brain areas via the contribution of gamma coherence to circuit and brain functioning (Fries, 2015). Although the exact mechanism of action of tACS is still under debate, the explicit aim of gamma-tACS is to restore disrupted endogenous gamma oscillations in AD patients. tACS is presumed to entrain or synchronize neuronal networks inducing changes in oscillatory brain activity (Antal & Paulus, 2013). Particularly, at the single neuron level, tACS would polarize neurons with alternating polarities, resulting in a subthreshold resonance (Aspart et al., 2018). On the other hand, spike synchrony of converging neurons, which is enhanced by tACS, can enhance information transfer and speed at a network level (Butts et al., 2007).

Accordingly, improvements of cognitive functions in AD patients (and mice models of AD) have been attributed to the enhancement of information flow and integration due to improved gamma coherence and E-I balance (Liu et al., 2022). Cross-frequency coupling and phase synchronization are neural interactions indicative of information gating and communication within and between broad networks during cognitive processes (Roux & Uhlhaas, 2014; Siegel et al., 2012). This neuronal coherence is observed not only across neuronal networks and brain regions (Varela et al., 2001) but also between frequency bands (known as cross-frequency coupling) having a critical role in cognitive functioning (Engel et al., 2001; Uhlhaas & Singer, 2012). Recent work has suggested that gamma and theta waves show a phase-phase coupling with multiple gamma cycles associated with every single cycle of theta. Gamma and theta waves are aligned in a way that gamma waves always begin at the same relative phase within a theta wave (Scheffer-Teixeira & Tort, 2016). Theta-gamma coupling underlies memory processes in the prefrontal-hippocampus pathway (Canolty & Knight, 2010; Engel et al., 2001). Moreover, the severity of memory impairment observed in MCI and AD patients is predicted by the disruption of theta-gamma coupling (Goodman et al., 2018; Moretti et al., 2011; Musaeus et al., 2020). Therefore the cognitive improvement observed after gamma-tACS in AD patients could be due to the effect of gamma entrainment on the E-I imbalance and disturbed theta-gamma coupling (Grover et al., 2021).

Interestingly, over the last few years, gamma entrainment has been shown to impact also microglia, astrocytes, and vasculature. Particularly, animal studies suggest that 40 Hz optogenetic and sensory stimulation attenuates AD-related neuropathology (i.e., reduction of extracellular Aβ and intracellular p-Tau aggregates) by inducing microglia responses. Due to the scarcity of empirical evidence, Adaikkan and Tsai (2020) proposed three possible mechanisms of interaction between gamma entrainment and glia activation. The first, because of its high ion (e.g., K+)-buffering capacity (Bellot-Saez et al., 2017), glia could take part in entrainment regulating ionic flow between the cortical layers (Adaikkan et al., 2019; Martorell et al., 2019) during prolonged gamma stimulation. Second, glia could exhibit an indirect response to gamma stimulation as its plasma membranes are exposed to high ionic fluctuations or are in physical contact with neurons. Third, glia response after gamma stimulation could be mediated by neuromodulatory systems (e.g., norepinephrine has been shown to regulate the dynamics of microglial surveillance) (Liu et al., 2019). Furthermore, gamma entrainment affects blood vessel dilation increasing CBF over the stimulated brain area (Chan et al., 2021; Sprugnoli et al., 2021). The release of factors such as vasodilators and vasoactive intestinal peptides, or morphological changes of neurovascular motifs, may explain the interaction between gamma entrainment and vessel dilatation (Adaikkan & Tsai, 2020; Deng & Jin, 2017). The improvement of glial functioning and cerebral blood flow can reduce Aβ accumulation, p-Tau aggregates, and brain atrophy. This knowledge about the cellular, circuit, and neurophysiological mechanisms of gamma activity comes from animal models, but the general principles of local neurocircuit mechanisms of gamma oscillations can be generalized to humans (Middleton et al., 2008).

**Fig. 5:**
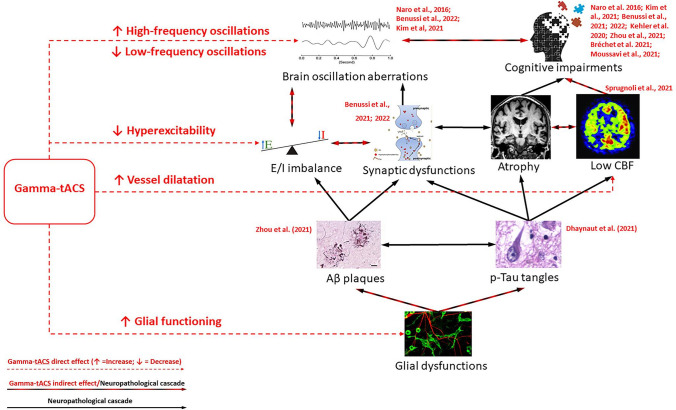
**Schematic representation of hypothesized gamma-tACS effects on the AD-related neuropathological cascade, with relevant references for each target effect.** Gamma-tACS (square on the left) directly affects (red arrows) brain oscillations, excitatory-inhibitory (E-I) balance, cerebral blood flow (CBF), and morpho-functional properties of microglia and astrocytes. In turn, such improvements can indirectly impact (black and red arrows) brain atrophy, amyloid-beta (Aβ) plaques, and neurofibrillary phosphorylated-tau (p-Tau) tangle formation, as well as cognitive functions. On the right side of the figure we reported the main neuropathological, markers of AD. The black (and black and red) arrows indicate the probable direction or sequence of AD neuropathological cascade

All the proposed mechanisms of action of gamma-tACS on AD neuropathology are summarized in Fig. 5. We point out that another neuromodulatory technique, i.e. sensory stimulation, can entrain gamma oscillations in AD patients for therapeutic purposes (Manippa et al., 2022). Indeed, the seminal study of Clements-Cortes and colleagues demonstrated that six 30-minute sessions of 40 Hz vibrotactile stimulation improve cognitive functioning in AD (Clements-Cortes et al., 2016). Since then, several studies showed that Non-invasive Gamma ENtrainment Using Sensory stimulation (GENUS), providing 40 Hz light or sound stimulation, can entrain rs-EEG gamma oscillations in healthy participants (Jones et al., 2001; Lee et al., 2021), reduce brain atrophy, and increase both functional connectivity and memory performance in AD patients (Chan et al., 2021; Cimenser et al., 2021). Similar preliminary results have been obtained with an at-home gamma sensory stimulator (Hempel et al., 2021; Williams et al., 2021). Two randomized clinical trials evaluating the long-term effects of GENUS on AD symptoms and neuropathology are currently ongoing (NCT04042922, NCT03556280) and will shed light on the actual therapeutic potential of this technique.

### Methodological Limitations

Taken together, the reviewed studies depict a favorable scenario of the suitability of gamma tACS as a therapeutic technique to treat dementia. However, some methodological issues should be highlighted. The limited number of studies, small sample sizes, modest statistical findings, and large variability of study procedures, targeted domains, and outcome measures make it hard to draw definitive conclusions regarding the most effective gamma-tACS protocols for MCI and AD treatment.

Specifically, sample size, recruitment criteria, and group arrangement represent potential issues for the generalization of results. For instance, the majority of the studies recruited around 20 participants, mainly AD or MCI patients, with two studies including different forms of dementia with the groups arranged according to arbitrary criteria (Kehler et al., 2020; Moussavi et al., 2021). Even more problematic is the lack of a control or placebo condition (i.e., sham treatment or group) in some studies, making interpretation of the tACS effects difficult. For instance, two studies compared the effect of combined cognitive training program and 40 Hz-tACS. Although tACS improved cognitive functioning more than the sole cognitive training, no tACS-only conditions were included in that study, leaving it unclear whether tACS alone would have had similar effects. From a statistical point of view, in some cases the reports were poor and the results modest.

Another important issue is the little adoption of cognitive tests having parallel or repeatable versions (adopted by Benussi et al., 2021, 2022) to avoid confusing learning and intervention effects. The use of tests with parallel versions such as RAVLT (Schmidt, 1996), or Repeatable Battery for the Assessment of Neuropsychological Status (RBANS; Randolph, 1998) should be strongly encouraged. Despite the lower clinical meaningfulness, ad-hoc editable tests (e.g., the computerized word-word association test and FNAT; Troyer et al., 2011) could be also adopted. Finally, we highlight the strong statistical limitations of the qualitative data provided by studies having a very small sample without a control condition (Bréchet et al., 2021; Dhaynaut et al., 2022).

In general, successful results from mice models still need to be translated into successful therapies for AD patients (Zahs & Ashe, 2010). Beyond the cognitive effects, the use of different neuropathological biomarkers in human and animal AD studies makes this transition particularly difficult. For instance, immunohistochemistry can be used as an outcome measure in mice models or human post-mortem studies only (Iaccarino et al., 2016; Martorell et al., 2019; Singer et al., 2018). The biomarkers adopted in some clinical trials (i.e., p-Tau and Aβ levels, microglia activity or morphology) can be evaluated in humans indirectly and with expensive and invasive techniques. Concerning this issue, the use of easily accessible and affordable blood-based biomarkers represents a promising alternative (Zetterberg & Bendlin, 2021). Furthermore, some authors have recently suggested that an indirect measure of cholinergic transmission such as TMS-elicited SAI, which is consistently observed to be reduced in MCI and AD patients, could be adopted together with biomarker measures (Benussi et al., 2018; Padovani et al., 2019). Benussi and coworkers demonstrated restoration of intracortical cholinergic and GABAergic neurotransmission after 60 min of 40 Hz-tACS over the medial parietal cortex or precuneus via SAI (Benussi et al., 2021, 2022). The effect of multi-session 40 Hz-tACS on SAI should be further investigated in future research. As shown by Naro and colleagues (2016), the use of EEG in conjunction with acute tACS could represent a valuable electrophysiological biomarker of disease progression. Finally, future studies should investigate the potential of personalized NIBS protocols: the adoption of computational modeling of electric fields allows the optimization of tACS montage and parameters for each patient, based on structural or functional neuroimaging, functional connectivity, or electrical brain activity (e.g., Miranda et al., 2018; Saturnino et al., 2019).

### Other NIBS for Dementia Treatment

A relevant number of studies tested the effect of other NIBS approaches on dementia-related cognitive and behavioral symptoms (Wang et al., 2020), particularly transcranial direct current stimulation (tDCS) and repetitive TMS (rTMS). TDCS is another transcranial electrical stimulation (tES) technique that involves the application of a weak direct electrical current through two or more electrodes placed on the scalp. It acts by modulating cortical excitability through hyperpolarization or depolarization of the resting membrane potential (Nitsche & Paulus, 2000). Prolonged stimulation induces long-term potentiation- and depression- like plasticity, involving the glutamatergic system (Stagg et al. 2018). rTMS involves the administration of trains of magnetic pulses applied over the scalp, causing localized electric currents via electromagnetic induction. The induced current elicits action potentials in neurons of the targeted region (Hallett, 2000). rTMS can be administered at a low frequency (lower than 5 Hz; LF-rTMS), high frequency (over 5 Hz; HF-rTMS), or in continuous and intermittent theta burst stimulation mode (cTBS and iTBS, respectively). HF-rTMS and iTBS induce excitatory effects, whereas LF-rTMS and cTBS induce inhibitory effects (Chervyakov et al., 2015). Similar to tDCS, rTMS induces long-term potentiation or depression-like plasticity (LTP or LTD). Thus although tDCS and rTMS work through different mechanisms, their primary physiological and behavioral effects are based on LTP or LTD-like changes of synaptic strength, rather than on modulations of oscillatory activity. Therefore, the mechanisms of action of these interventions differ qualitatively from those of tACS.

Although several studies have demonstrated cognitive improvement or stabilization in MCI and AD patients after tDCS (Liu et al., 2017), a recent meta-analysis (Inagawa et al., 2019) has questioned these findings due to the small sample sizes and the heterogeneity in stimulation parameters and cognitive assessments. On the other hand, a random effects analysis revealed that HF-rTMS over the left dlPFC and LF-rTMS over the right dlPFC significantly improve memory functions in MCI and AD patients (Chou et al., 2020). Moreover, HF-rTMS targeting the right inferior frontal gyrus of AD and MCI patients significantly enhanced executive performance, and the aftereffects of 5 to 30 consecutive rTMS sessions could last up to 4 to 12 weeks (Chou et al., 2020). To sum up, Hf-rTMS and anodal tDCS over the dlPFC seem to induce long-lasting effects in dementia patients (Šimko et al., 2022). One study has recently examined the effect of 40 Hz-rTMS on AD patients (Liu et al., 2022). Participants that received 12 sessions of bilateral 40 Hz-rTMS over the angular gyrus, as compared to sham, showed (a) improved cognitive functioning lasting for 8 weeks after the intervention, (b) gamma power entrainment in the temporo-parietal cortex, (c) reduced grey matter volume loss and, (d) improved brain functional connectivity. Despite the widely accepted mechanism for neural effects of rTMS is the alteration of synaptic plasticity through LTP or LTD (Ma et al., 2014), this study demonstrates that specific rTMS protocols can regulate ongoing oscillatory activity in the gamma frequency band (Brignani et al., 2008; Thut et al., 2011), but likely with less lasting after-effects compared with tACS (e.g., Hosseinian et al., 2021).

Besides the different mechanisms of action, tES (e.g. tACS and tDCS) offers several advantages over rTMS in terms of cost, portability, and practicability. Indeed, home-based stimulation that can be self-administered by patients or caregivers (i.e., enhancing intervention feasibility), is only possible with transcranial electrical or sensory stimulation devices at present (Bréchet et al., 2021). Moreover, the higher rates and severity of adverse effects of rTMS compared to tACS should be considered, especially in vulnerable populations (Matsumoto & Ugawa, 2017; Stultz et al., 2020). While transient headache, local scalp discomfort, and phosphenes (more frequent with frontal montages) have been described for both techniques, these side effects are more frequent after rTMS than tES (Krishnan et al., 2015). As for tES, very rare skin lesions after long-lasting high-frequency or high-intensity rTMS have been described, while dizziness (in case of posterior stimulation) and very rare cases of seizures occurred during or after rTMS (Stultz et al., 2020). Accordingly, in our reviewed studies, participants reported no serious adverse effects during and after gamma-tACS, even after multiple sessions.

### The Stimulation Frequency Issue

All tACS studies targeting AD patients focused on gamma oscillations, particularly 40 Hz. This choice originates from current knowledge about the key role of theta-gamma coupling in memory processes and AD progression (Goodman et al., 2018; Lisman & Jensen, 2013), the observed effect of 40 Hz stimulation in mice models of AD, and rsEEG evidence in AD patients. Indeed AD rsEEG is characterized by a widespread slowing of spectral density, including a decrease in gamma, but also alpha and beta bands, and an increase in theta oscillations (Cassani et al., 2018; Cecchetti et al., 2021). In addition, a decrease in the complexity of electrical brain activity and neuronal synchronization has been reported, with evidence for cortical connectivity reduction (Babiloni et al., 2016, 2020; Blinowska et al., 2017). Interestingly, some studies show increased alpha band synchronization (López et al., 2014), as a compensatory mechanism (Nakamura et al., 2018). However, it remains unclear whether this effect extends to memory enhancement in healthy samples.

Booth et al. (2021) systematically reviewed tACS studies targeting working memory and long-term memory (LTM, including declarative and episodic memory) in healthy adults. Both these memory systems are impaired from the early stages of AD (Petersen et al., 2014). This review suggests a reliable site- and frequency-dependent effect of tACS on memory performance. Particularly, strong evidence suggests that posterior theta-tACS improves working memory performance, whilst enhancement of LTM can be achieved by anterior gamma-tACS (Grover et al., 2022). Studies on alpha- and beta-tACS showed, instead, inconclusive or null results. In addition, Booth et al. (2022) highlighted a correspondence between the effects of tACS on LTM and working memory performance and the electrophysiological outcome (gamma and theta entrainment, respectively). In line with their observations, a recent study on older healthy adults reported selective improvements in working memory and LTM through dissociable spatiospectral entrainment of brain rhythms. Grover et al. (2022) showed that selective changes in working memory and LTM functions were obtained via inferior parietal lobe theta-tACS and dlPFC gamma-tACS, respectively. Such improvements were sustained for at least 1 month after the intervention. The endogenous increase of theta and decrease of gamma oscillations in AD patients discourages the use of theta-tACS in favor of gamma-tACS, as the former is assumed to be dysfunctional. To the best of our knowledge, no study investigated the potential therapeutic role of alpha and beta-tACS in AD, although these frequency bands are disrupted from the early stages of the disease (Cecchetti et al., 2021; Koelewijn et al., 2017). Interestingly, compared to tACS studies in dementia patients, the most common frequency of stimulation to target LTM in healthy adults is 60 Hz (Grover et al., 2022; Nomura et al., 2019). Particularly, Javadi et al. (2017) showed that the effects of gamma-tACS on declarative memory, rather than on a specific frequency, depends on matching between the stimulation frequency administered during the encoding and the retrieval phase of the task (i.e., either 60 or 90 Hz). When mismatched gamma-tACS was administered during these two stages (e.g., 60 Hz during encoding and 90 Hz during retrieval), no memory improvement was observed. This is in line with the hypothesis that successful memory retrieval derives from the reinstatement of neural activity patterns that occur during encoding (Javadi et al., 2017; Nomura et al., 2019).

The abovementioned findings leave an open question. Is 40 Hz the only valuable and effective stimulation frequency for AD treatment? Following the findings observed in mice models of AD, research in patients exclusively focused on 40 Hz stimulation. However, not all available evidence in the current review demonstrates successful gamma entrainment in AD patients after 40 Hz-tACS interventions, including effectiveness on neuropathology. In line with the literature on healthy individuals, Naro et al. (2016) obtained enhanced gamma oscillations and cognitive processing in AD patients using a gamma-tACS protocol with frequencies randomly changing between 40 and 120 Hz. Even sensory stimulation studies are casting doubts about the most effective stimulation frequency to entrain gamma oscillations. For instance, Lee et al. (2021) showed that lights flickering at 34 to 38 Hz entrained gamma oscillations stronger than flickering at 40 to 50 Hz in young adults, whereas 32 or 34 Hz light flickering induces stronger gamma entrainment than 38 or 40 Hz flickering in older adults (Park et al., 2022). Further, Khachatryan et al. (2022) found that the inclusion of a cognitive task during the GENUS session exerts positive effects on the strength and extent of gamma oscillations, promoting the propagation of gamma entrainment to deep brain areas. Similar methodological investigations on gamma-tACS effectiveness for gamma entrainment have not been conducted in AD patients yet. Future methodological studies should shed light on the optimization of tACS parameters and protocols, to find the best way to entrain gamma activity, and improve therapeutic effects in dementia patients.

## Conclusion

Dementia has a huge economic and social impact. The total annual cost per person with dementia is on average €32,506.73 in Europe and €42,898.65 in the United States. This means that dementia is a “trillion-dollar” disease (Cantarero-Prieto et al., 2020). Epidemiologists estimate that approximately 100 million people worldwide will have AD in 2050 (World Alzheimer Report, 2015), and no effective and established treatments are available for slowing dementia progression or preventing MCI to AD conversion (Cooper et al., 2013). The development of reliable, feasible, and cost-effective treatments to delay or avoid MCI conversion to AD is a primary goal for future research. Together with the investigation of acute effects, future clinical trials could focus on the long-term effects of multi-session gamma-tACS protocols on cognitive, neurophysiological, and neuropathological biomarkers of MCI due to AD. 

Our systematic review shows that gamma-tACS protocols are safe and well-tolerated non-pharmacological treatments for AD and MCI with promising perspectives for the improvement of memory functions and EEG low-to-high band ratio. Their effect on neuropathological biomarkers and, more in general, on dementia prognosis should be further investigated. There is also a great need to investigate further outcomes of interventions, such as brain connectivity, brain morphometry, and neuropathology markers, to corroborate the hypothesized mechanisms of action. In this context, an extension of follow-up measures, which are of restricted duration (i.e., not longer than 3 months in the available studies) might be ideal, as well as the arrangement of booster sessions that can be scheduled throughout disease progression, and might be suited to prolong effects. Also, further studies are required to optimize gamma-tACS parameters of stimulation. In summary, we believe that greater effort should be put into this progressing field of research, for advancing AD diagnosis, prognosis, and treatment with the final goal to prevent MCI to dementia conversion, or slowing down the neuropathological dementia cascade with the final goal to ameliorate patients’ wellbeing.

## Data Availability

Not applicable.
