# Correction to: Cognitive and Neuropathophysiological Outcomes of Gamma-tACS in Dementia: A Systematic Review

**DOI:** 10.1007/s11065-025-09676-4

**Published:** 2026-02-26

**Authors:** Valerio Manippa, Annalisa Palmisano, Michael A. Nitsche, Marco Filardi, Davide Vilella, Giancarlo Logroscino, Davide Rivolta

**Affiliations:** 1 Department of Education, Psychology and Communication, University of Bari “Aldo Moro”, Bari, Italy; 2 Department of Psychology and Neurosciences, Leibniz Research Centre for Working Environment and Human Factors, Dortmund, Germany; 3 Department of Neurology, University Medical Hospital Bergmannsheil, Bochum, Germany; 4 Center for Neurodegenerative Diseases and the Aging Brain, University of Bari “Aldo Moro” at Pia Fondazione “Cardinale G. Panico”, Tricase, Lecce Italy; 5 Department of Basic Medicine, Neuroscience and Sense Organs, University of Bari “Aldo Moro”, Bari, Italy

**Correction to: Neuropsychology Review (2023) 34:338–361**

10.1007/s11065-023-09589-0

In the originally published version of this article, the heading for the “Results” section was inadvertently omitted. This starts with the sentence: “Ten studies (273 patients analyzed) published between 2016 and 2022 were included in this review (Table 1)” within the Methods subheading “Data Extraction and Risk of Bias”. It includes the content under the subheadings “Single-session studies” and “Multi-session studies,” and continues until the start of the “Discussion” section.

The original article has been corrected.

